# Effects of Inflammatory Response Genes on the Immune Microenvironment in Colorectal Cancer

**DOI:** 10.3389/fgene.2022.886949

**Published:** 2022-04-08

**Authors:** YaChen Wang, Luping Zhang, Guanghuan Shi, Mingqing Liu, Weidan Zhao, Yingli Zhang, Ying Wang, Nan Zhang

**Affiliations:** Department of Gastroenterology, The First Hospital of Jilin University, Changchun, China

**Keywords:** inflammatory response, tumor microenvironment, colorectal cancer, immune cell infiltration, risk score

## Abstract

**Background:** The close relationship between colorectal cancer and inflammation has been widely reported. However, the relationship between colorectal cancer and inflammation at the genetic level is not fully understood.

**Method:** From a genetic perspective, this study explored the relationship between inflammation-related genes and the immune microenvironment in colorectal cancer. We identified prognostic genes, namely *CX3CL1*, *CCL22*, *SERPINE1*, *LTB4R*, *XCL1*, GAL, *TIMP1*, *ADIPOQ*, and *CRH*, by using univariate and multivariate regression analyses. A risk scoring model for inflammatory response was established, and patients in The Cancer Genome Atlas (TCGA) database and Gene Expression Omnibus (GEO) database were divided into two groups: high risk group and low risk group.

**Results:** The analysis showed that the prognosis of the two groups was significantly different, and the low-risk group had a higher survival rate and longer survival time. Pathways related to apoptosis, inflammatory response, and hypoxia were significantly enriched as shown *via* Gene Set Enrichment Analysis (GSEA). Activated dendritic cell infiltration was found in both the TCGA and GEO databases, and the *CCL21* gene played a significant role in the process of activated dendritic cell infiltration. *CCL21* gene was also positively correlated with inflammatory response, and the gene expression and risk score were significantly different between the two groups.

**Conclusion:** In summary, inflammatory response has a direct impact on patients with colorectal cancer in the prognosis and immune infiltration and further research studies on the inflammatory response can help in advancing the development of immunotherapy for colorectal cancer.

## Introduction

Colorectal cancer is the third most common cancer in the world (Stoffel and Yurgelun, 2016). It is also one of the main causes of cancer deaths in both men and women globally (Drewes et al., 2016). The possible link between inflammation and tumors was first revealed in the 19th century by Rudolf Virchow (Hooper et al., 2012). Epidemiological and clinical studies have also shown that patients who have Crohn’s disease and ulcerative colitis, the two major types of inflammatory bowel disease, are at increased risk of colorectal cancer (CRC) (Rizzo et al., 2011). The gut is particularly rich in human microbes, and bacteria disrupt the homeostasis by activating immune signaling pathways, leading to an inflammatory environment (Brennan and Garrett, 2016). Tumor microenvironment plays an important role in the growth and development of tumors (Esteva et al., 2019; Strasser and Birnleitner, 2019). Inflammation can cause the aggregation and activation of immune cells, and the activated immune cells promote the proliferation of tumor cells by secreting pro-inflammatory cytokines and chemokines (Ferrari et al., 2019). Therefore, the relationship between inflammatory response and the immune microenvironment of colorectal cancer has attracted much attention. In this study, we screened out the inflammatory response genes related to the prognosis of intestinal tumors and constructed an inflammatory response risk score model. Enrichment of the model-related pathways was examined. Furthermore, we screened out the genes which may influence immune cell infiltration using the inflammatory response risk score model. The purpose of this study was to examine the relationship between inflammatory response and intestinal tumor immune microenvironment at the genetic level.

## Materials and Methods

### Data Sources

RNA sequences and clinical data of relevance in this study were obtained from the TCGA and GEO (GSE39582) databases. GSE39582 contains the largest sample size of CRC patients with the most complete clinical information.

### Construction of the PPI Network

We used a String database to construct the PPI network of genes related to inflammatory response.

### Construction and Grouping of Inflammatory Response Models

We downloaded the genes related to inflammatory response, and then screened for the genes associated with the prognosis of colorectal cancer. The expression level of each gene in the TCGA and GEO databases was multiplied by the expression coefficient, followed by calculation of the risk score for each patient. For further analyses, the patients in the two databases were divided into high and low risk groups according to the median value of the risk score obtained from the TCGA database.

### Survival Analysis

There were 445 patients in the TCGA database, and the follow-up time was 12 years. There were 562 patients in the GEO database who were followed for 16 years. The “survival” and “SurvMiner” packages from R (4.0.3) language were used to analyze the prognosis of these patients. Kaplan-Meier method was used to draw the survival curves, and the log-rank test was used to test the statistical significance. *p* < 0.05 was considered significant.

### ROC Curve Analysis

The 1-, 3-, and 5-years survival rates of patients in two databases were analyzed. The “survival,” “SurvMiner,” and “timeROC” packages from R (4.0.3) language were used to analyze and calculate the area under the ROC curve (AUC). If the area under the curve of 1-, 3-, and 5-years survival rates gradually increases and exceeds 0.5, it indicates that the model has a high accuracy for predicting the survival of patients. The survival of the two groups was represented by a risk column and risk curve.

### Heat Map

The heatmap representing gene expression in this experiment was drawn by the “PheatMap” package of R (4.0.3) language.

### Cox Regression Analysis

Survival kit of R (4.0.3) language was used to analyze the inflammatory response genes that were significantly correlated with prognosis, and age, sex, T, N, M, and risk score were used for single-factor and multi-factor prognostic analyses.

### Gene Set Enrichment Analysis

We downloaded and extracted the genes associated with inflammation from the 1 Gene Set Enrichment Analysis (GSEA) website. Gene sets with NOM *p* < 0.05 and FDR *q* < 0.06 were considered to have statistical significance.

### Analysis of the Correlation Between Genes and Inflammatory Response

We downloaded the genes involved in regulating immune cells and screened for the ones that play a crucial role in inflammatory response. The “ggplot2,” “GGPUBR,” and “ggExtra” packages of R (4.0.3) language were then used to analyze the correlation between these genes and inflammatory response, as well as their expression between the high and low risk groups.

## Results

### Extraction and Screening of Inflammatory Response-Related Genes

**FIGURE 1:**
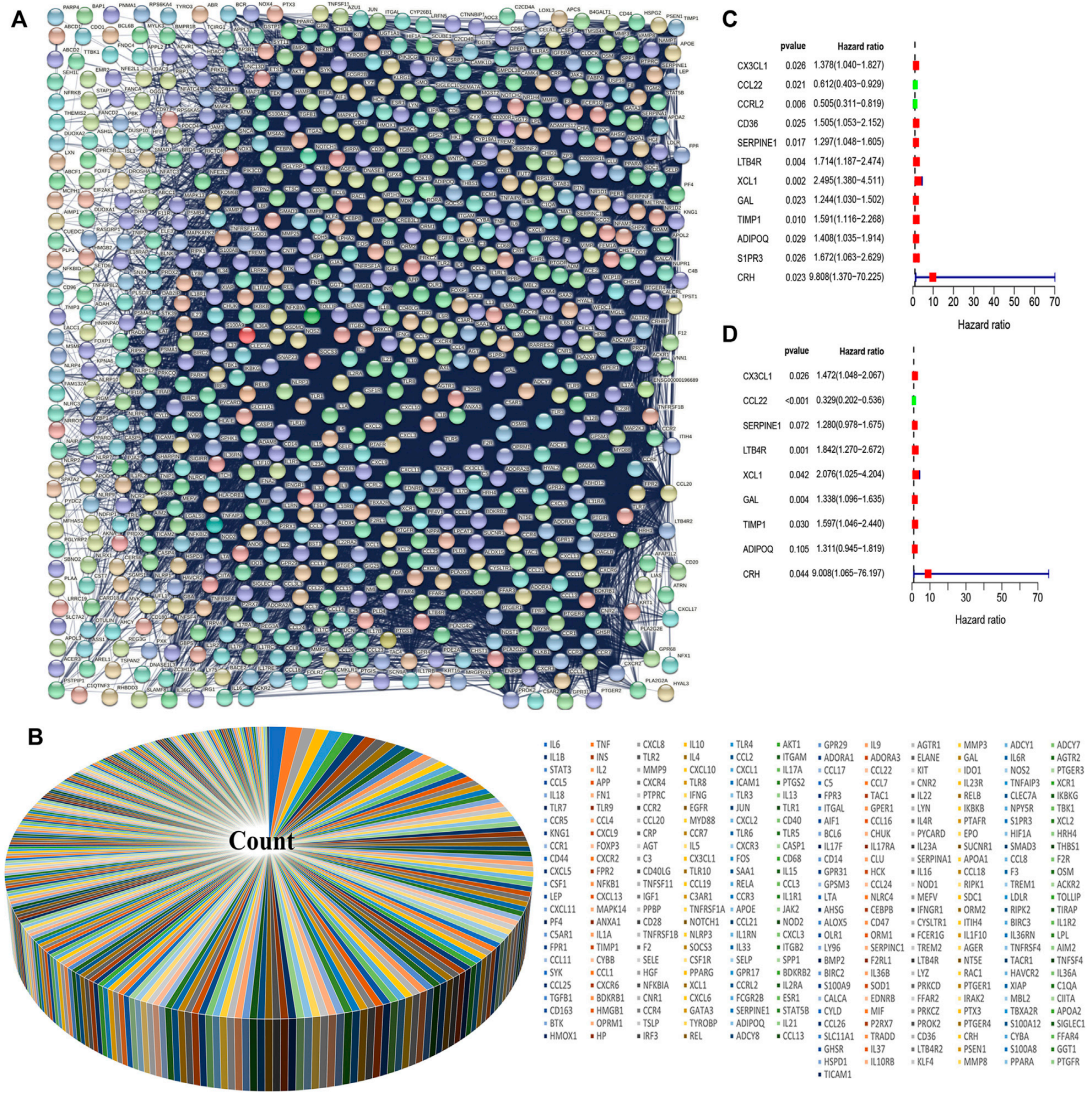
Screening of inflammatory response genes and their influence on prognosis. **(A)** Protein interaction network of inflammatory response genes. **(B)** The 301 inflammatory response genes with the largest number of adjacent nodes have been represented by a pie chart. **(C)** The genes with *p* value <0.03 were screened by univariate Cox regression analysis. **(D)** Multivariate Cox regression analysis was performed to select the inflammatory response genes that can independently affect the prognosis.

We downloaded the Ontology gene set from the Gene Set Enrichment Analysis (GSEA) website and extracted the inflammatory response-related genes from the data set. We used the STRING protein-protein interaction (PPI) to establish the relationship between the proteins of the inflammatory response-related genes (Figure 1A). Because of the large number of genes related to the inflammatory response, we selected 301 genes which were the largest number of adjacent nodes for subsequent gene screening (Figure 1B). Next, we downloaded gene expression data and clinical information of intestinal tumors from the Cancer Genome Atlas (TCGA) database and extracted the expression levels of inflammatory response-related genes. Cox univariate analysis was used to screen out genes related to CRC prognosis (Figure 1C). Since there were several genes related to prognosis, we selected the genes with *p* < 0.03 for subsequent modeling. *CCL22* and *CCRL2* were identified as low risk genes, while *CX3CL1*, *CD36*, *SERPINE1*, *LTB4*, *XCL1*, *GAL*, *TIMP1*, *ADIPOQ*, *S1PR3*, and *CRH* were identified high-risk genes. We included these genes in subsequent Cox multivariate regression analysis. The final prognosis model was constructed using *CX3CL1*, *CCL22*, *SERPINE1*, *LTB4R*, *XCL1*, *GAL*, *TIMP1*, *ADIPOQ*, *CRH*, and *CCL22* which was a low-risk gene (Figure 1D).

### Effects of Inflammatory Response-Related Genes on Prognosis

Multivariate Cox regression analysis revealed nine inflammatory response genes which were related to the prognosis of colorectal cancer and were subsequently used to construct the prognosis model. The risk score for each patient was obtained by multiplying the amount of gene expression by the corresponding regression coefficients (0.3864, −1.111, 0.2468, 0.6110, 0.7304, 0.2915, 0.4683, 0.2706, and 2.198 for *CX3CL1*, *CCL22*, *SERPINE1*, *LTB4R*, *XCL1*, *GAL*, *TIMP1*, *ADIPOQ*, and *CRH*, respectively). The median risk score of the patients in the TCGA and GEO databases as the standard, the patients were divided into high and low-risk groups.

**FIGURE 2:**
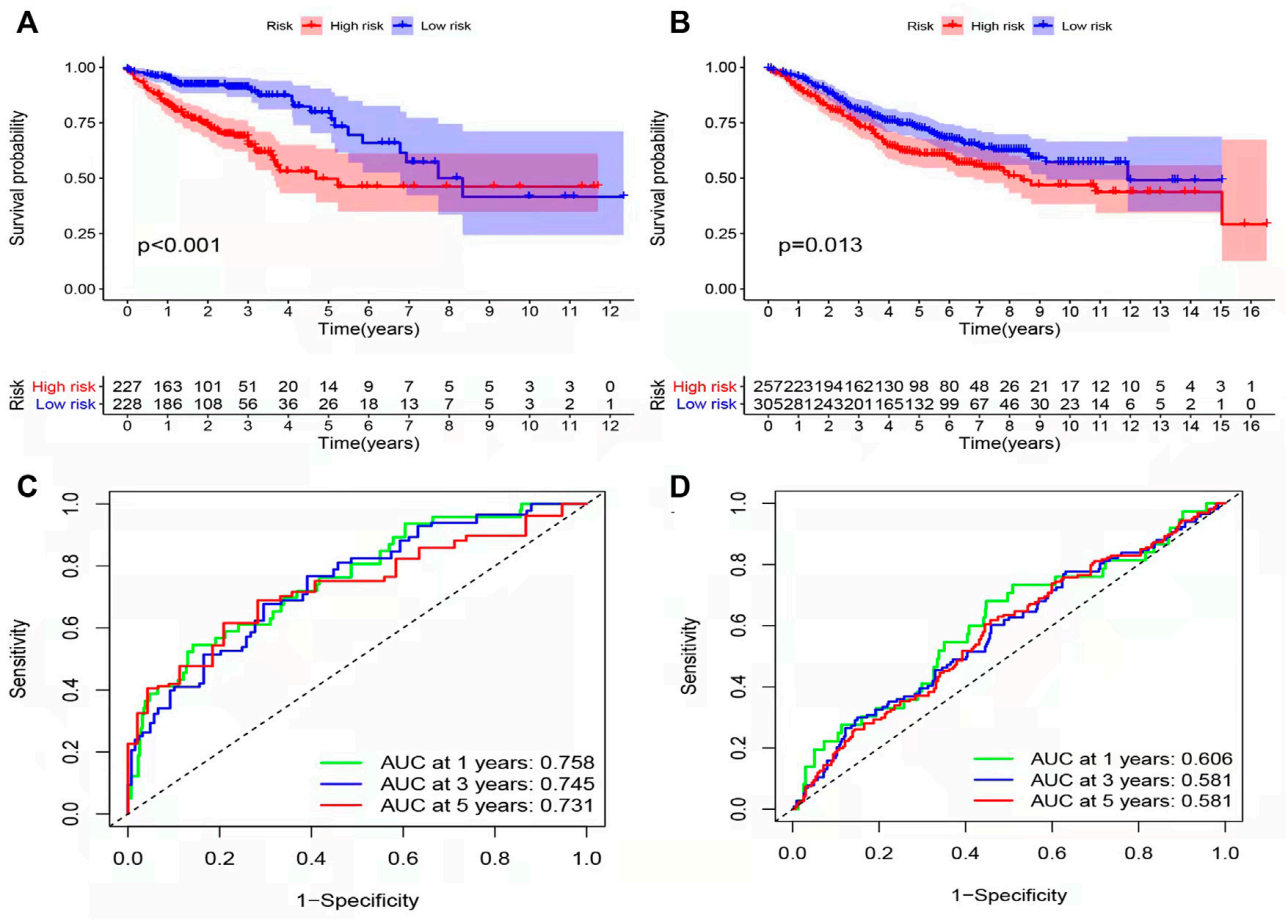
Effect of the model on prognosis. **(A,B)** Patients with colorectal cancer were divided into high-risk group and low risk group using Kaplan Meier method (TCGA, GEO). The log-rank test was used to compare the survival time between the high-risk and low-risk groups (*p* values were less than 0.001 and equal to 0.013, respectively). **(C,D)** By using ROC curve to evaluate the accuracy of the prediction model. The area under the ROC curve of TCGA and GEO databases was greater than 0.5.

**FIGURE 3:**
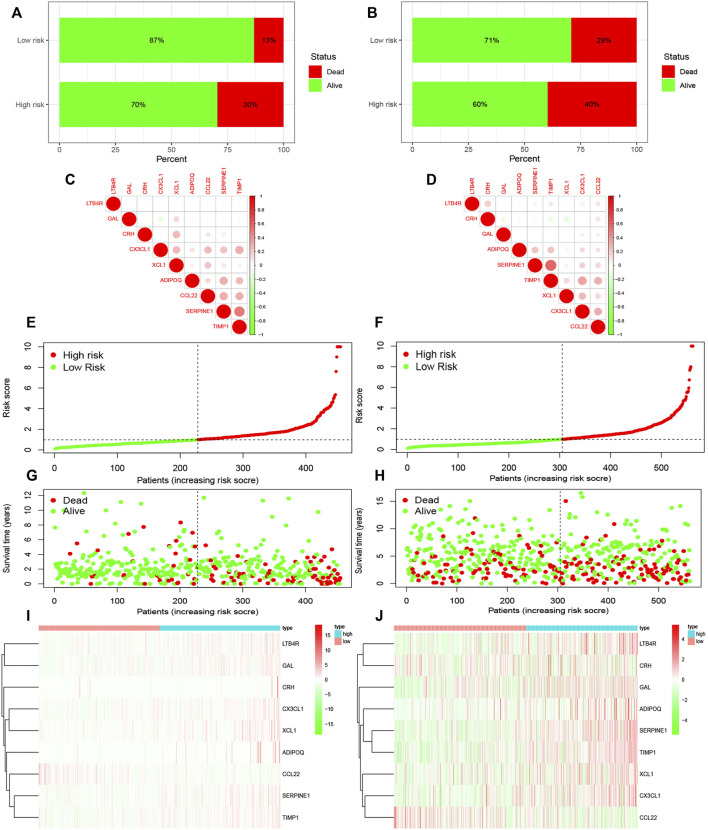
Survival rate and prognosis of patients using the model, along with the expression of genes in the model in the high-risk and low-risk groups. **(A,B)** Survival rates in the TCGA and GEO databases. **(C,D)** Relationship between the genes in the model. **(E,F)** Risk scores of patients in the TCGA and GEO databases. **(G,H)** Survival time of patients in the high and low risk groups in the TCGA and GEO databases. **(I,J)** Expression of the genes in the model in the high and low risk groups of the TCGA and GEO databases.

Results of survival analysis for the two groups of patients is shown in Figures 2A, B. Significant differences in the survival were observed between the high-risk and low-risk groups (*p* < 0.05). The area under the receiver operating characteristic (ROC) curve of the TCGA database and GEO database was greater than 0.05 (Figures 2C, D). However, the area under the curve did not increase with the increase in follow-up time for either the TCGA or GEO databases. This shows that the prediction model has some issues regarding the accuracy of prognosis and needs improvement. In order to observe the survival of the patients in the high and low risk groups more intuitively, we used a risk histogram to display the survival status of patients in the TCGA and GEO databases (Figures 3A, B). Patients in the high-risk group had lower survival rates than those in the low-risk group, indicating that our model can distinguish the high-risk group from the low-risk group. Next, we analyzed the relationship between the prognosis-related inflammatory response genes in the model (Figures 3C, D). The relationship between the risk score and survival rate of patients is illustrated by risk curves in Figure 3 (E and F plotted the risk scores of the patients in the high and low risk groups, respectively). Patients in the medium-high risk group lived shorter lives than those in the low-risk group (Figures 3G, H), and with increasing time, the number of deaths also decreased. Finally, by using thermography, we evaluated the expression of the genes in the high and low risk groups in the model (Figures 3I, J).

### Effect of Different Clinical Characteristics on the Prognosis of CRC

**FIGURE 4:**
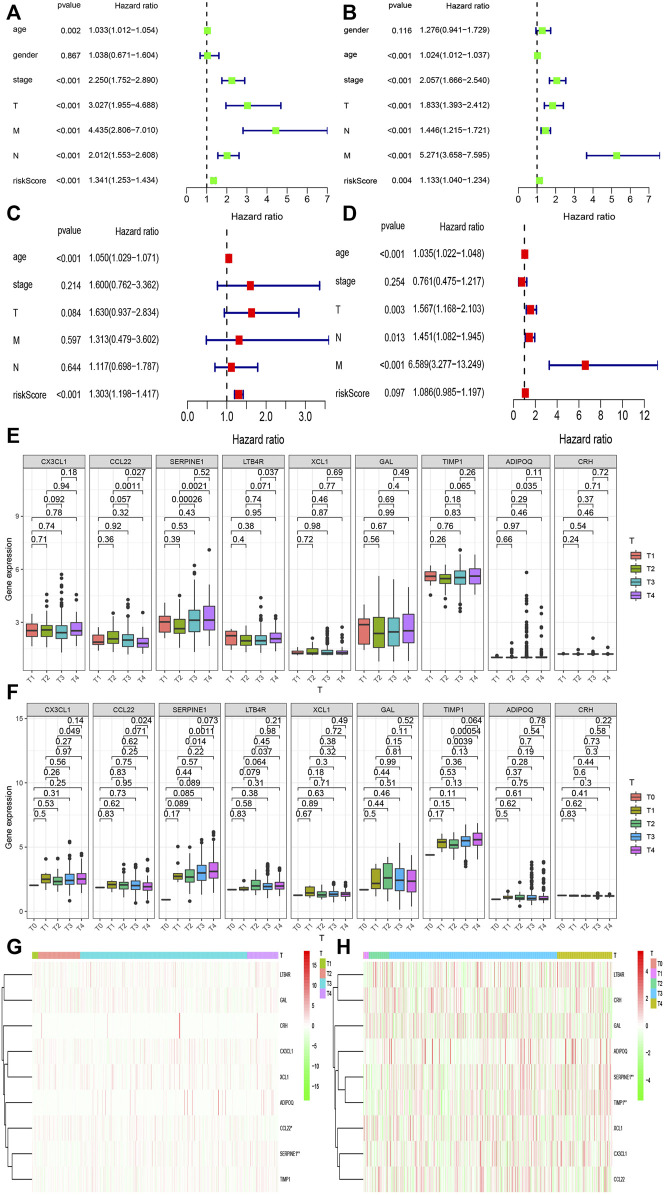
Relationship between the risk model and different clinical traits. **(A,B)** Univariate prognostic analysis was performed on age, sex, T, N, M stage, and risk score of colorectal cancer patients in the TCGA and GEO databases. **(C,D)** Multivariate prognostic analysis was performed on age, sex, T, N, M stage, and risk score of colorectal cancer patients in the TCGA and GEO databases. **(E,F)** Expression of genes between among T stages in the TCGA and GEO databases. **(G,H)** Heat maps of the expression of inflammatory response genes among different T stages in models in the TCGA and GEO databases.

Different clinical characteristics have different effects on the prognosis of patients with CRC. Along with the risk score obtained from the model, we analyzed the influence of different clinical characteristics on the prognosis of patients with CRC. First, Cox univariate regression analysis was used to determine the association between clinical features and the prognosis of patients from the two databases (Figures 4A, B). Sex had no effect on the prognosis of patients, whereas other factors had an impact on the prognosis and were all high-risk factors. The risk score of the prediction model in the TCGA and GEO databases was also less than 0.05, which indicates that the risk score is related to prognosis of patients. Multivariate regression analysis of these factors showed that age, T, M, N, and risk score were all significant independent prognostic factors (*p* < 0.05) (Figures 4C, D). Next, we observed that there was a difference in the expression of inflammatory response-related genes between different T stages (Figures 4E, F). We found a significant difference in the expression of *SERPINE1* between different T stages (*p* < 0.05). Figures 4G, H are thermograms demonstrating the expression of the inflammatory response-related genes between different T stages in the two databases.

### Enrichment of Inflammation Related Gene Pathways in the High and Low Risk Groups

**FIGURE 5:**
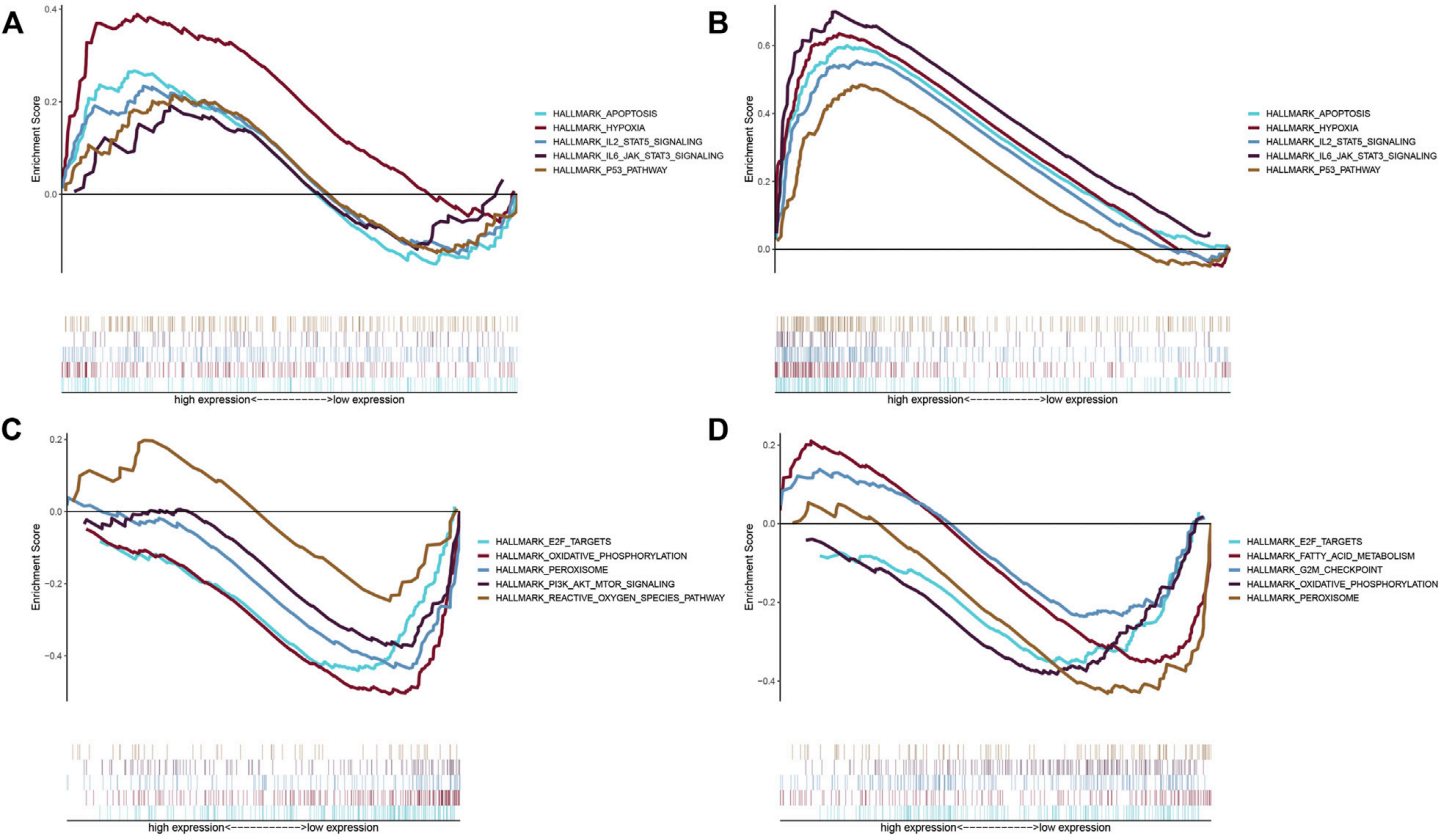
Enrichment of pathways in the high-risk and low-risk groups. **(A)** Enriched gene sets in the hallmark gene collection by the high-risk score in the TCGA database. The lines with different colors represent different gene sets. The up-regulated genes are located near the origin of the coordinate on the left, while the down-regulated genes are located on the right side of the *x*-axis. Gene sets with NOM *p* < 0.05 and FDR *q* < 0.06 were statistically significant. Only a few major gene sets are shown. **(B)** Enriched gene sets in the hallmark collection by the high-risk score in the GEO database. Gene sets with NOM *p* < 0.05 and FDR *q* < 0.06 were statistically significant. Only a few major gene sets are shown. **(C)** Enriched gene sets in the hallmark collection by the low-risk score in the TCGA database. Only gene sets with NOM *p* < 0.05 and FDR *q* < 0.06 were considered statistically significant. Only a few major gene sets are shown. **(D)** Enriched gene sets in the hallmark collection by the low-risk score in the GEO database. Gene sets with NOM *p* < 0.05 and FDR *q* < 0.06 were statistically significant. Only a few major gene sets are shown.

There were also differences in the enrichment of inflammatory response-related genes between the high and low risk groups. In order to understand the enrichment of pathways, GSEA software was used to analyze the pathways in the two risk groups. The high-risk groups in the TCGA and GEO databases demonstrated enrichment of a large number of apoptotic, hypoxia, and inflammation related pathways, including apoptosis, hypoxia, IL-2-STAT5 signaling, IL-6-JAK-STAT3- signaling, and the P53-pathway (Figures 5A,B). The enriched pathways in the low-risk group were mostly related to oxidative phosphorylation and peroxidation including E2F-target, oxidative-phosphorylation, peroxisomes, PI3K-AKT-mTOR signaling, and reactive-oxygen-species pathways (Figures 5C,D).

### Infiltration of Immune Cells

**FIGURE 6:**
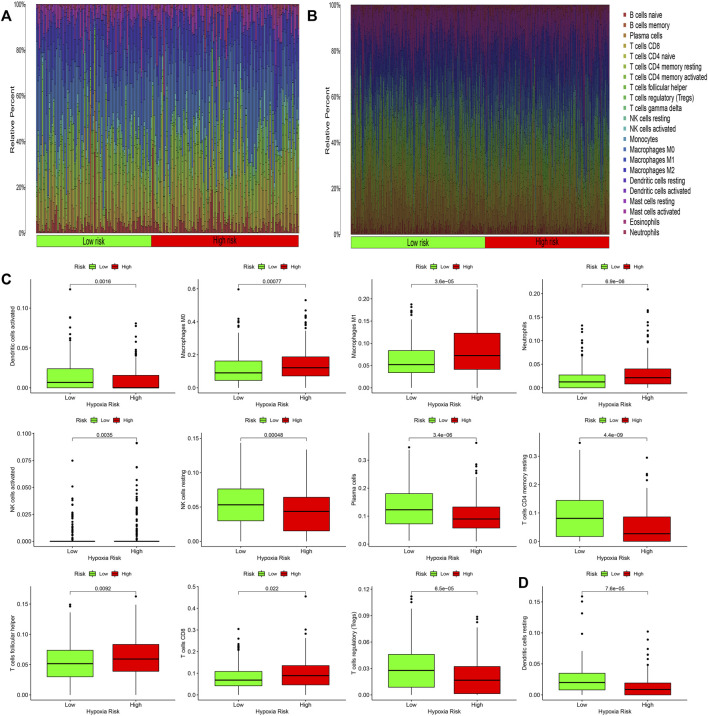
Immune cell infiltration in high and low risk groups. **(A,B)** Thermography of inflammatory response-related gene risk scores and immune cell infiltration in the TCGA and GEO databases. **(C,D)** Infiltrating immune cells in the TCGA and GEO databases were significantly associated with the risk scores.

**FIGURE 7:**
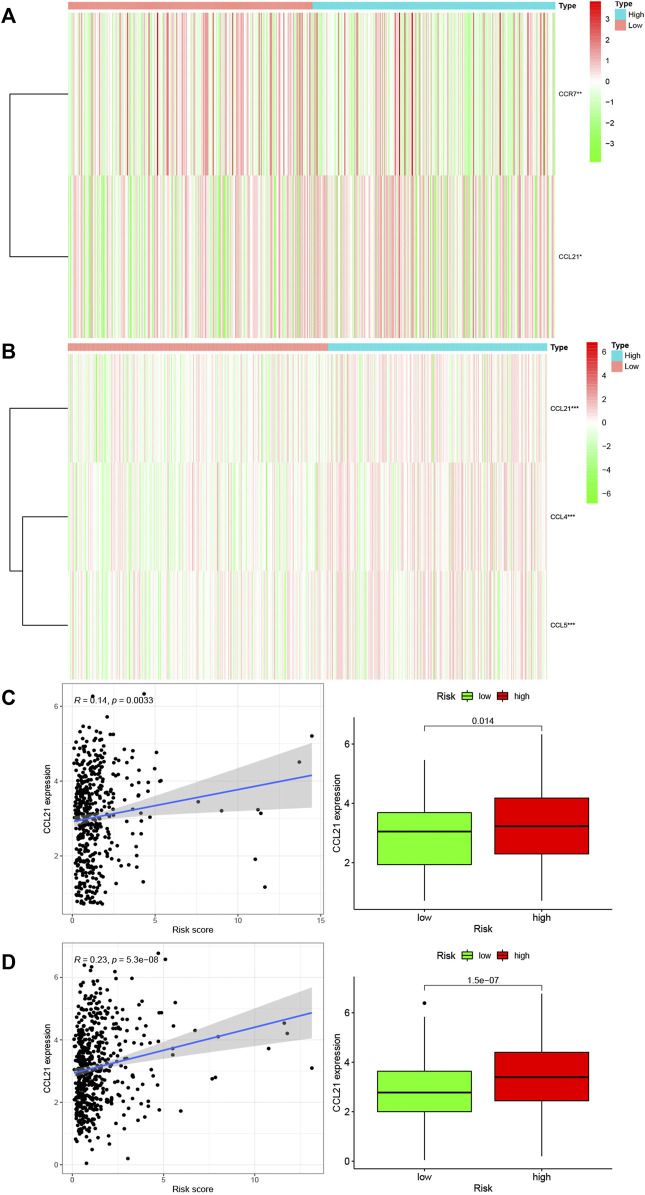
Relationship between immune cell regulatory genes and risk scores of genes associated with inflammatory response. **(A,B)** Heat maps of the expression of genes significantly related to immune cell regulation in different risk groups in TCGA and GEO databases (*p < 0.05; **p < 0.01; ***p < 0.001). **(C,D)** Correlation between CCL21 gene significantly associated with immune cell regulation and risk score in the TCGA and GEO databases. The blue line in the figure fitted the linear model between gene expression and risk score of genes related to inflammatory response, and Pearson’s coefficient was used to test the correlation. Box chart showed the difference in CCL21 gene expression between groups with high and low risk of inflammatory response related genes (*p* < 0.05).

Inflammation often causes the infiltration of immune cells. Therefore, we studied the infiltration of immune cells in each risk group in the TCGA and GEO databases. Figures 6A, B respectively show the infiltration of immune cells in the high-risk groups and low-risk groups. In the TCGA database, the infiltration of 11 kinds of immune cells was significantly different between the high-risk and low-risk groups (Figure 6C, *p* < 0.05). In the GEO database, the infiltration of one kind of immune cell was different between the high-risk and low-risk groups (Figure 6D, *p* < 0.05). Activated dendritic cells had different infiltration in the two databases. We downloaded immune related genes from the website of Tracking Tumor Immunophenotype and screened out the genes that regulate the activation of dendritic cells. We created heat maps (Figures 7A, B) to show the expression of genes regulating the infiltration of dendritic cells in the TCGA and GEO databases in the high and low risk groups. The expression of the *CCL21* gene was significantly different between the two databases (*p* < 0.05). A correlation curve between *CCL21* gene expression and the risk score was drawn, which revealed that *CCL21* gene expression was positively correlated with the risk score. Additionally, the expression of *CCL21* gene was different between the high-risk and low-risk groups (Figures 7C, D).

## Discussion

During tumor development, changes observed in the tumor sites resemble chronic inflammation, a process described as “a tumor is an unhealed wound” that promotes tumor survival (Grivennikov et al., 2010). Studies have shown that chronic inflammation is the leading cause of many cancers in humans (Bernstein et al., 2001). Although inflammation can be used as a strategy against microbes, it is also thought to be a marker of cancer and plays a key role in tumorigenesis. Inflammatory response plays a decisive role in different stages of tumor progression, including initiation, promotion, malignant transformation, invasion, and metastasis (Grivennikov et al., 2010), and can promote carcinogenesis by inducing gene mutations, stimulating angiogenesis and cell proliferation, or inhibiting cell apoptosis (Rutter et al., 2004). Inflammation-related genes are involved in these processes and act on corresponding pathways or regulate immune cells. Our study screened the inflammatory response genes associated with CRC and identified the core genes, among which *CX3CL1*, *CCL22*, *SERPINE1*, *LTB4R*, *XCL1*, *GAL*, *TIMP1*, *ADIPOQ*, and *CRH* were closely related to the prognosis of patients. *CX3CL1* can induce the ERK pathway and cell proliferation, and also plays a specific tumor promoter role in breast cancer expressing *ERBB2* (Tardáguila et al., 2013). *CCL22* is a chemokine that is highly expressed in tumors, and promotes tumor growth, in addition to playing a role in tumor-related immunosuppression (Wiedemann et al., 2016). *SERPINE1* and *TIMP1* promote the migration and invasion of tumor cells (Song et al., 2016a; Klimczak-Bitner et al., 2016). *SERPINE1* may also promote the invasion and metastasis of colorectal cancer (Simone et al., 2015). *LTB4R* is a potent chemoattractant involved in inflammatory and immune responses to the paeoniflora-like signaling pathway (Wilson et al., 2014), which is involved in all inflammatory diseases (Peters-Golden and Henderson, 2007). *XCL1* promotes antitumor activity (Chou et al., 2020), and *XCL1* expression is also significantly related to the number of tumor infiltrating CD8+T cells as well as the expression of *PD-L1* in tumor cells (Tamura and Yoshihara, 2020). *GAL* methylation status may be an important marker for predicting clinical prognosis in patients who are with head and neck squamous cell carcinoma (Misawa et al., 2017). *ADIPOQ* gene play a role in chronic inflammation and cancer (Divella et al., 2017). *CRH* expression is associated with the advanced stage of ovarian cancer (Minas et al., 2007). It can be seen that these inflammatory response-related genes are closely related to tumors. Accordingly, we chose these nine genes to build an inflammatory model.

Inflammatory responses are associated with poor prognosis in a variety of tumors (Minas et al., 2007; Sano et al., 2018; Zhang et al., 2019). In order to further examine the relationship between the model and prognosis of patients, we evaluated the prognosis of patients by taking the product of the expression level and expression coefficient of the nine genes in the model in the two databases as the risk score. Significant differences in prognosis were found in the high-risk and low-risk groups, and patients in the low-risk group lived significantly longer than those in the high-risk group. However, in the process of using ROC curve to evaluate the accuracy of survival analysis, we found that the AUC value did not change much with the extension of time in the TCGA and GEO databases. This indicates that the accuracy of the ROC curve to evaluate survival was not ideal, which may be related to the following factors: First, the survival rate of CRC is 73.8% (70.0% for rectal cancer, 75.9% for colon cancer) (Fatemi et al., 2015), five- year survival rate is 68.4% (Kong et al., 2019), and average survival time is 142.17 ± 21.60 months (Fatemi et al., 2015). However, we evaluated 1-, 3-, and 5-years survival. Therefore, the survival situation could not be accurately reflected. Second, the sample was relatively small. Third, TCGA and GEO databases were selected for this study, and the predictive and prognostic accuracy of this model needs to be verified using multiple databases. Lastly, adjuvant therapies such as surgery and chemoradiotherapy also have an impact on the prognosis of patients (Fatemi et al., 2015). This study analyzed the impact of age, sex, T, M, N stage, and risk score on the prognosis of patients, and found that the risk score had a corresponding impact on the prognosis of patients in both databases. However, in the multivariate analysis, the *p* value of the risk score in the GEO database was >0.05, indicating that the risk score could not be used as an independent prognostic factor. On further examination, we found that all colorectal cancer types in the GEO database were adenocarcinomas, and the samples were all from France. Because of the limitations of tumor types and sample sources, the results may not accurately reflect the effect of inflammatory response on prognosis of colorectal cancer patients. Some patients had received chemotherapy, and the prognosis of CRC patients is related to the depth of tumor invasion, presence of lymph node metastasis (Haq et al., 2009), presence of other diseases, presence of venous or lymphatic invasion, tumor grade (Zlobec and Lugli, 2008), and genetic factors. These factors were not taken into account in this study, which is a limitation.

Furthermore, by using GSEA enrichment analysis to study the pathway enrichment in the high-risk groups and low-risk groups in the two databases. The study showed that the enriched pathways were mostly associated with hypoxia, inflammatory factors, and apoptosis.

Hypoxia and inflammation are closely related (Biddlestone et al., 2015). The inflammatory environment itself tends to be hypoxic (Watts and Walmsley, 2019), possibly because the metabolically active cells such as neutrophils migrating from the peripheral blood to the inflammatory tissue consume a large amount of energy (Borregaard and Herlin, 1982; Pollard and Borisy, 2003), and increase the oxygen demand (Rao and Suvas, 2019). Moreover, inflammation often leads to activation of the cellular hypoxia response pathways (Liu et al., 2014). Infection activates keratinocytes, macrophages, dendritic cells, and other cells, leading to the production of inflammatory cytokines (Wong and Goeddel, 1986; Mooney et al., 1990). Hypoxia also increases circulating proinflammatory cytokine levels (Song et al., 2016b). Tumor development has been shown to be associated with the inactivation of apoptosis (Xu et al., 2009). The downregulation of the tumor suppressor gene p53 can lead to reduced cell apoptosis and promote tumor growth (Bauer and Helfand, 2006), which are associated with many cancers (Rodrigues et al., 1990; Gasco et al., 2002). Some scientists also believe that apoptosis drives the proliferation and metastasis of tumor cells (Wang et al., 2013). Thus, there is a close relationship between inflammatory response, inflammatory factors, and apoptosis.

The immune system plays a decisive role in the initial inflammatory response to infection and injury and is the main driver of the inflammatory protective response (Carrillo-Salinas et al., 2019). Therefore, the inflammatory response often leads to the infiltration of immune cells. In our study, the infiltration of 11 types of immune cells including activated dendritic cells, macrophages M0, macrophages M1, neutrophils, activated natural killer (NK) cells, NK cells, plasma cells, CD4 memory T cells, helper T cells, CD8 T cells, and regulatory T cells was significantly differently between the high-risk and low-risk groups in the TCGA database. However, only the infiltrates of activated dendritic cells in the GEO database were significantly different in the high and low risk groups. Subsequently, the genes regulating activated dendritic cells were screened, and it was found that the expression of *CCL2* gene was different in the groups with high and low risk in the TCGA and GEO databases. Other studies have also shown that the *CCL2* gene is associated with inflammatory responses. *CCL2* is a chemokine that attracts and activates monocytes (Conti and Rollins, 2004). *CCL2* plays a crucial role in tumor cell growth, metastasis, and host immune response (Zhuang et al., 2018). Additionally, *CCL2* has been shown to have both tumor stimulating and antitumor effects. Recent studies have suggested that *CCL2* plays a major role in tumor progression and metastasis (Li et al., 2013). *CCL2* can enhance the migration and invasion ability of prostate cancer cells (Natsagdorj and Izumi, 2019), as well as induce the invasion of liver cancer cells (Zhuang et al., 2018). High levels of *CCL2* expression in various types of tumors are also associated with poor prognosis (Yang et al., 2016); for example, the increased level of *CCL2* expression is related to poor prognosis in breast cancer patients (Lebrecht et al., 2004; Fang et al., 2015). Although many studies have elaborated the relationship between inflammatory response and colorectal cancer from the perspective of genetics and pharmacology, few articles have explored the relationship between inflammatory response-related genes and CRC at the genetic level. This study analyzed the relationship between inflammatory response-related genes and colorectal cancer at the genetic level, which can facilitate further research on colorectal cancer.

However, this study only carried out bioinformatics correlation analysis and did not explore the specific mechanism of inflammation response genes affecting prognosis. This study only proved that the risk scoring model established by us was related to prognosis of patients with colorectal cancer patients. Therefore, prospective studies, such as some basic and clinical studies, are needed to explore the specific mechanisms by which the genes we have identified interact with colorectal cancer.

In conclusion, inflammatory response plays a significant role in the prognosis of CRC patients and in the tumor immune microenvironment. Understanding the relationship between inflammatory response and immune cells is conducive to the faster application of effective immunotherapy for CRC treatment in the clinic, leading to an improvement in the prognosis of colorectal cancer patients.

## Data Availability

Publicly available datasets were analyzed in this study. This data can be found here: https://portal.gdc.cancer.gov/ and GEO (GSE39582) database.

## Abbreviations

AUC, area under the ROC curve; CRC, colorectal cancer; GEO, gene expression omnibus; GSEA, gene set enrichment analysis; NK, natural killer; PPI, protein-protein interaction; TCGA: the cancer genome atlas.
